# A New Dental Quarterly—A New Degree in Dentistry

**Published:** 1877-10

**Authors:** 


*A New Dental Quarterly—A New Degree in Dentistry.*—Prof. Henry S. Chase, of St Louis, Mo., who for the past year has edited the *Missouri Dental Journal*, announces his withdrawal from the conduct of that Magazine, which is resumed by Drs. Judd and Eames.

At the same time is announced the forthcoming of a *Dental Quarterly*, to be edited by Drs. Chase and Spaulding. The industry and talents of Dr. Chase will doubtless make this one of the most valuable of our exchanges.

The catalogue of another new Dental College—the "Western

College of Dental Surgeons"—is received, located in St Louis: Dr. C. W. Spaulding, Dean. A new feature instituted is the establishment of the degree of "Master of Mechanical Dentistry,' the candidate for this degree to pass examination in Anatomy, Chemistry and Mechanical Dentistry.

The prospectus announces in substance that any one who has earned his degree without the aid of schools is as much entitled to adiploma as those who spend their time in the lecture halls of a college.

nnovations of established usages are too frequent to be startling in this day, but it really seems hardly fair to add to the stock of titles which the one specialty of Dentistry offers, and it can scarcely place us in a more favorable light before the public, professional or unprofessional. We have now in addition to the established D. D. S., M. D. S., (Master of Dental Surgery,) L. D. S., (Licentiate of Dental Surgery,) D. M. D,, (Dr. of Dental Medicine,)—and now comes M. M. D. Are there others still? It certainly suggests a want of uniformity in the minds of those instituting degrees as to what the title of a dentist should be. Who can now blame those who put all sorts of rank and title on their signs cards, &c., even to a publication of the order of knighthood!

As to the graduation of applicants who have passed satisfactory examinations, but who studied elsewhere than in dental colleges, it occurs to us that in this day of numerous and accessible schools, located in almost every section of the country, and with all the facilities which they offer for thorough education, it hardly seems a time to take a step which looks like offering a premium to young men to stay away from those institutions, as this seems to do, seeing that they are offered graduation on examination as to qualifications. All who know the subject are fully convinced that, a dental college is, and a private office is not, the place to study dentistry, and while it may possibly be true that occasionally a man is found who from stress of circumstances or other causes cannot attend lectures. but who has thoroughly qualified himself at home for practice, the exceptions are too few to establish such a precedent as this. Besides, the practical qualifications which would entitle one to graduation on these terms would undoubtedly enable their possessor to make his practice sufficiently lucrative to be able to spare the time for lectures.

We are not in a position as a profession, and the colleges are not in a condition to justify a step that will look like a lowering of the standard of requirements on the part of those desiring to enter its ranks. We repeat that dental education is so cheap and the schools so available as to give no excuse to those who really wish to render themselves thorough in theory and practice. It cannot bring good but harm as we see it, to the profession, to education, and to those who are the recipients of the degrees.

